# Vaccine pricing strategies in China

**DOI:** 10.1136/bmjgh-2022-011405

**Published:** 2023-07-04

**Authors:** Haonan Zhang, Bryan Patenaude, Chao Ma, Hai Fang

**Affiliations:** 1 China Center for Health Development Studies, Peking University, Beijing, China; 2 Department of Health Policy and Management, Schoolf of Public Health, Peking University, Beijing, China; 3 Department of International Health, Johns Hopkins University Bloomberg School of Public Health, Baltimore, Maryland, USA; 4 International Vaccine Access Center, Bloomberg School of Public Health, Johns Hopkins University, Baltimore, Maryland, USA; 5 National Immunization Program, Chinese Center for Disease Control and Prevention, Beijing, China; 6 Peking University Health Science Center - Chinese Center for Disease Control and Prevention Joint Research Center for Vaccine Economics, Peking University, Beijing, China

**Keywords:** Vaccines, Health economics, Health policy

**Summary box**

- Pricing strategies for National Immunisation Programme (NIP) vaccines and non-NIP vaccines in China are quite different.
- Prices of NIP vaccines are low, and this pricing strategy may not fit for including non-NIP vaccines into NIP.
- Non-NIP vaccines are relatively more expensive, and their prices are expected to be reduced through volume-based procurement to meet the need of citizens.

## Standfirst

Fang *et al* argue that China has a huge and unique vaccine market and appropriate pricing strategies for vaccines play an important role in determining National Immunisation Programme (NIP), vaccine access and coverage.

Vaccines are a vital public health success in protecting human health, having dramatically reduced the morbidity and mortality of infectious diseases. The prices of vaccines play a significant role in determining their accessibility. The pricing strategy of vaccines refers to the method and mechanism used by manufacturers, government agencies and health providers to jointly determine the price of vaccines in the market. In China, the vaccine market is enormous, with over 700 million vaccine doses produced in 2019 and a market size that reached CNY75 billion in 2020.^1 2^ The vaccine industry and immunisation programmes in China exhibit unique characteristics that distinguish them from those in other developed or developing countries. Vaccines in China are classified into two categories based on their inclusion in the NIP: NIP vaccines and non-NIP vaccines. The dynamic, multicategory structure of the vaccine market in China has resulted in different pricing strategies for NIP and non-NIP vaccines. The pricing strategies for vaccines in China are critical in determining public access to both NIP and non-NIP vaccines. NIP vaccines are often priced very low due to the limited budget for national procurement. In contrast, non-NIP vaccines in China are typically priced relatively high since individuals pay for them out of pocket.

## Overview of the vaccine market in China

According to the Vaccine Administration Law of the People’s Republic of China,^3^ NIP vaccines are defined as vaccines provided free of charge by the government and are administered to eligible citizens. Non-NIP vaccines are those that are not yet included in the NIP and are available to citizens at their own expense and/or on a voluntary basis. The NIP was launched in 1978 in China, and the programme initially included four vaccines: BCG vaccine, oral poliovirus vaccine, diphtheria-tetanus-pertussis (DTP) vaccine and measles vaccine. Hepatitis B vaccine was further included in 2002. The NIP in China was expanded to include hepatitis A (HepA), Measles-Mumps-Rubella, Japanese encephalitis and meningococcal AC vaccines in 2008. The NIP also includes vaccines against hantavirus (that causes haemorrhagic fever with renal syndrome), anthrax and leptospirosis in epidemic areas or selected population groups. Currently, the list of NIP includes 14 vaccines against 15 diseases.^4^ The National Health Commission takes responsibility for the NIP and established the National Immunisation Advisory Committee to make evidence-based decisions about incorporating vaccines into the NIP. Combination vaccines, such as Pentacel, in China are classified as non-NIP vaccines, even though they contain NIP vaccines.

The coverage rate of all NIP vaccines exceeds 90% in China.^5^ Non-NIP vaccines have a much lower coverage rate compared with the universal national coverage of NIP vaccines. In 2019, the coverage rate of non-NIP vaccines ranged from 1.8% for the third dose of rotavirus vaccine, 5.1% for the third dose of 13-valent *Pneumococcal* conjugate vaccine (PCV13), 25.0% for the third dose of *Haemophilus influenzae* type b (Hib) vaccine and 67.1% for the first dose of varicella vaccine. There were significant inequalities in non-NIP vaccination, which were strongly associated with family incomes.^6^

## Pricing strategies of NIP vaccines

From the perspective of general pricing strategies,^7^ NIP vaccines are targeted at all eligible citizens and are positioned closely as a public good.^8^ Prices of NIP vaccines are strictly regulated in China, and procurement prices have been kept at a very low level (table 1). The Chinese Center for Disease Control and Prevention (CDC) uses the National Central Vaccine Procurement to purchase all NIP vaccines in China once per year.^9^ This volume-based procurement process involves a bidding system where prices and doses are simultaneously set for selected manufacturers for the entire country. Usually, two manufacturers with the lowest bidding prices are selected to supply each NIP vaccine. In China, the government does not provide direct subsidies to manufactures for vaccine procurement. Instead, it invests in and subsidises a few manufacturers that supply NIP vaccines.

**Table 1:** Prices of selected NIP vaccines in China compared with prices in USA and UNICEF

Vaccines (US dollars in 2021)	Prices for NIP in China (prices calculated by PPP)	Prices for CDC in the USA	Prices for private sector in the USA	Prices for UNICEF
Hepatitis B	1.22 (1.89)	16.27	26.34	0.24–0.70
BCG	0.76 (1.17)	–	–	0.10–0.28
Polio, inactivated (IPV)	5.43 (8.36)	15.11	38.74	1.50–4.11
Polio, attenuated (bivalent)	0.42 (0.65)	–	–	0.11–0.19
Diphtheria, tetanus and pertussis	0.89 (1.37)	19.88–20.37	27.03–34.15	0.17–0.19
Diphtheria and tetanus	0.10 (0.15)	–	–	0.17–0.18
Measles, mumps and rubella	3.84 (5.92)	23.67	89.87	1.55–4.47
Meningococcal A polysaccharide (MPV-A)	0.22 (0.34)	–	–	–
MPV-AC	1.85 (2.85)	–	–	–
Japanese encephalitis	1.52 (2.34)	–	–	0.45
Hepatitis A, attenuated	4.55 (7.01)	–	–	–

Exchange rate: US$1=CNY6.4515 in 2021.

PPP：US$1=CNY4.187 in 2021.

Data source: Prices for NIP in China provided by China CDC, prices for CDC and private sector in the USA from CDC vaccine price list,^11^ prices for UNICEF from UNICEF vaccines pricing data.^10^

CDC, Centers for Disease Control and Prevention; NIP, National Immunisation Programme; PPP, purchasing power parity.

The prices of NIP vaccines in China, whether in nominal US dollars or purchasing power parity, were comparable to those set by UNICEF. However, they were significantly lower than the prices charged for the CDC and private sector in the USA.^10 11^ Despite the low pricing strategy of NIP vaccines allowing for universal coverage with limited budgets, a major limitation is that newly developed vaccines such as PCV13, rotavirus and Hib cannot be introduced into NIP due to their high prices in China.

## Pricing strategies of non-NIP vaccines

The primary goal of non-NIP vaccine manufacturers when setting prices is to maximise profit, which is determined by considering both marginal cost and marginal revenue. The pricing strategy of non-NIP vaccines is influenced by factors such as the elasticity of demand and the level of competition among manufacturers.^12^ The pricing strategy for non-NIP vaccines in China is quite different from that of NIP vaccines. The procurement of non-NIP vaccines is carried out by provincial and county CDCs, and the vaccine prices vary little from province to province. While there may be many manufacturers or suppliers for the same vaccine, price competition is limited due to the different vaccine procurement mechanism. The procurement process involves two stages. Prices and doses are procured separately for non-NIP vaccines. As the first stage, the provincial CDC request bidding for procurement prices of many non-NIP vaccines without specifying quantities of doses, and the same non-NIP vaccine can be supplied by many manufacturers with different prices. Manufacturers do not have strong incentives to reduce prices of non-NIP vaccines at this stage, as they do not know how many doses can be sold. As the second stage, each county CDC actually procures non-NIP vaccines with a relatively small number of doses, and any manufacturer on the procurement list of the provincial CDC can be chosen. As a result, pricing for non-NIP vaccines is not as fiercely competitive as it is for NIP vaccines. However, the high prices of some non-NIP vaccines have led to very low coverage rates, which is a significant limitation.

The procurement prices of selected non-NIP vaccines in China are presented in table 2. The prices of non-NIP vaccines from both domestic and foreign manufactures in China were much higher than those set by UNICEF, but similar to or even higher than the prices charged for the CDC and private sector in the USA.^10 11^ Prices of some non-NIP vaccines in China may be higher or lower than those in the US CDC due to competition among vaccine manufacturers. If there is only a single manufacturer in the market, such as for Shingles, vaccine prices are often similar to or higher than those in the US CDC. However, when domestic manufacturers enter a market, such as with PCV13, they often set a lower price than existing foreign manufacturers and quickly gain a higher market share. As a result, foreign and domestic vaccine manufacturers in China adopt different pricing strategies. Foreign vaccine manufacturers import newly developed vaccines into China’s market with high prices, while domestic vaccine manufacturers often enter the market with lower prices. Foreign vaccine manufacturers have higher costs as their vaccine products are often imported, but their vaccines often face few competitors, such as with Shingles and 5-valent rotavirus vaccines. The competition introduced by the presence of more domestic manufacturers might contribute to the decrease in vaccine prices, such as 23-valent pneumococcal polysaccharide vaccine and PCV13.^13^ It is still possible to gradually reduce prices of non-NIP vaccines through the volume-based procurement,^14^ referring to the similar procurement of NIP vaccines.

**Table 2:** Prices of selected non-NIP vaccines in China compared with prices in USA and UNICEF

Vaccines (US dollars in 2021)	Prices by foreign manufacturers in China (prices calculated by PPP)	Prices by domestic manufacturers in China (price calculated by PPP)	Prices for CDC in the USA	Prices for private sector in the USA	Prices for UNICEF
Human papillomavirus (quadrivalent)	125.55 (MSD) (193.46)	–	–	–	4.50 (GAVI) 13.50–26.75 (MICs)
Human papillomavirus (9 valent)	201.19 (MSD) (310.01)	–	208.05	268.77	–
Rotavirus (5 valent)	45.47 (MSD) (69.98)	–	76.95	93.19	0.80–0.95
Pneumococcal polysaccharide vaccine (23 valent)	40.30 (MSD) (62.10)	34.72 (53.50)	65.8	117.08	–
Hepatitis A, inactivated	33.02 (MSD) (50.87)	20.15–26.82 (31.05–41.32)	22.64	36.66	7.55
Influenza (trivalent)	8.53 (Sanofi) (13.14)	7.13–9.30 (10.99–14.33)	–	–	–
Haemophilus influenzae type b	17.17 (Sanofi) (26.46)	17.67–19.07 (27.23–29.38)	10.39	18.24	–
DTaP-IPV-Hib (Pentacel)	94.55 (Sanofi) (145.69)	–	65.77	106.18	–
Pneumococcal conjugate (13 valent)	109.74 (Pfizer) (169.09)	73.32–95.02 (112.97–146.41)	158.18	226.43	2.90–3.30 (GAVI) 15.67–25.00 (MICs)
Hepatitis B	22.94 (GSK) (35.35)	19.07 (29.38)	16.27	26.34	0.24–0.70
Shingles	247.69 (GSK) (381.66)	–	104.53	171.57	–

Exchange rate: US$1=CNY6.4515 in 2021.

PPP: US$1=CNY4.187 in 2021.

Data source: Prices for non-NIP in China collected from procurement prices publicised by provinces, prices for CDC and private sector in the US from CDC vaccine price list,^11^ prices for UNICEF from UNICEF vaccines pricing data.^10^

CDC, Centers for Disease Control and Prevention; GAVI, Global Alliance for Vaccines and Immunization; MICs, middle-income countries; NIP, National Immunisation Programme; PPP, purchasing power parity.

## Towards efficient and fair pricing

Overpricing of vaccines raises health expenditure, hinders access to vaccines and weakens the effectiveness of disease prevention, while underpricing of vaccines discourages manufacturers from investing in the development of new vaccines. Vaccine pricing needs to balance the profitability of manufacturers and the health needs of the public. Globally, group procurement is commonly used to purchase vaccines in developed countries or by international organisations. In the USA, vaccine purchase groups (VPGs) serve a large number of medical practices in the private sector and often sign exclusive agreements with only one vaccine manufacturer.^15^ VPG agreements specify ‘product loyalty’ amounts of doses that the VPG and its members must purchase to receive discounted vaccine pricing. The US CDC signs contracts with a few vaccine manufacturers (only one or two) for one vaccine, and the purchase contracts for vaccines are for immunisation programmes that receive CDC immunisation cooperative agreement funds. The US CDC contract prices of vaccines are much lower than those offered by private sectors.^10^ GAVI and UNICEF try to lower vaccine prices by pooling procurement with assured funding and offering multiyear contracts.^16^ International experiences have shown that lower prices require a specified number of contracted doses for procuring vaccines, which has been employed for procurement of NIP vaccines but not for procurement of non-NIP vaccines in China.

The high prices of non-NIP vaccines continue to result in unmet demand in the Chinese market. A survey conducted in Shanghai, China revealed that factors such as income, education and convenience influence the acceptability of non-NIP vaccines. The survey also found that half of the participants who would not have opted for non-NIP vaccines would change their decision if non-NIP vaccines were covered by health insurance.^17^ The largest factor contributing to inequality in non-NIP vaccines is monthly household income per capita.^6^ Moreover, the procurement situation varies across provinces, and different types of vaccines are offered. To meet the health needs of the population and to improve the equity of vaccines, negotiations could be conducted at the national level to reduce vaccine prices through high-volume procurement and upfront investment in research and development. Typically, there are no government investments or subsidies available for non-NIP vaccines in China. However, some local governments have started direct subsidy programmes for individuals to receive human papillomavirus and/or influenza vaccination.

The inner value is central to vaccine pricing and requires extensive evidence to assess, for instance, clinical trials on vaccine efficacy and vaccine safety, economic evaluations and the willingness to pay of consumers. However, China’s NIP and pricing strategies have not yet adopted this approach. The selection of NIP vaccines and the introduction of new vaccines into the NIP have not been based on their inner value. Prices of NIP vaccines are very low, and while this has allowed China to vaccinate a vast population with high coverage rates using limited funding, it has resulted in low profitability for manufacturers and a lack of incentive to improve NIP vaccines or invest in new vaccine research and development. The absence of comprehensive evidence on the social benefits of vaccines may lead to underinvestment by governments, but this has limited impact on vaccine pricing as enterprises prioritise profit maximisation and governments base decisions on financial capacity. Additionally, some WHO-recommended vaccines, such as PCV13, rotavirus and Hib, have not yet been included in China’s NIP, resulting in low coverage rates and significant inequalities.

Prices of NIP vaccines could be set higher to incentivise manufacturers to improve vaccine quality and introduce new vaccines into the NIP. The National Central Vaccine Procurement could be used to procure non-NIP vaccines, which would significantly lower the prices of such vaccines. The vaccine bidding process of both NIP and non-NIP vaccines should simultaneously set both prices and doses procured. Only when manufacturers have strong incentives to lower their bidding prices due to high economics of scale resulting from a high number of doses, can prices of non-NIP vaccines be effectively reduced.

## Data Availability

The data that support the analysis of this study can be found in the article. All publicly available data used in the writing of this article are listed and cited in the text and reference list.
